# PGPR-Mediated Plant Immunity: From Microbial Recognition to Epigenetic Priming

**DOI:** 10.3390/plants15091368

**Published:** 2026-04-30

**Authors:** Dilek Unal, Shahlo Satimova, Durdigul Botirova, Murad Muhammad, Dilfuza Egamberdieva

**Affiliations:** 1 Department of Molecular Biology and Genetics, Faculty of Science, University of Bilecik Seyh Edebali, 11000 Bilecik, Türkiye; dilek.unal@bilecik.edu.tr; 2 Institute of Fundamental and Applied Research, Tashkent Institute of Irrigation and Agricultural Mechanization Engineers, Tashkent 100000, Uzbekistan; shahlosatimova93@gmail.com; 3 Faculty of Biology, National University of Uzbekistan, Tashkent 100174, Uzbekistan; durdigulbotirova@gmail.com; 4 State Key Laboratory of Desert and Oasis Ecology, Key Laboratory of Ecological Safety and Sustainable Development in Arid Lands, Xinjiang Institute of Ecology and Geography, Chinese Academy of Sciences, Urumqi 830011, China; muradbotany1@uop.edu.pk; 5 University of Chinese Academy of Sciences, Beijing 100049, China; 6 International Center for Strategic Development and Research (ISCAD), Ministry of Agriculture of Uzbekistan, Tashkent 100140, Uzbekistan

**Keywords:** biocontrol, chromatin remodeling, epigenetics, ISR, plant immunity, PGPR

## Abstract

The increasing demand for sustainable agriculture has intensified interest in beneficial microbes as eco-friendly alternatives to chemical pesticides for plant disease control. Among these, plant growth-promoting rhizobacteria (PGPR) have attracted great interest because they can suppress plant pathogens and strengthen plant health through molecular mechanisms. Recent studies suggest that PGPR protect plants from disease not only by directly attacking pathogens but also by changing how plant immune genes are expressed through epigenetic processes. This review brings together current knowledge on epigenetic regulation in plant–PGPR interactions, focusing on DNA methylation, histone modifications, and non-coding RNA pathways. PGPR colonization activates plant immune signaling through pattern recognition receptors, MAPK cascades, reactive oxygen species, and plant hormones. The review also covers the range of bacterial signals—including lipopolysaccharides, flagellin, cyclic lipopeptides, and volatile organic compounds—that prepare plant defenses, and explains how the recognition of these signals reshapes chromatin structure at defense genes. In addition, the review discusses how these changes may influence induced systemic resistance and examines emerging, though still limited, evidence on whether they could potentially be transmitted to subsequent generations. A better understanding of how microbial signals regulate host epigenetics may reveal new ways to improve plant immunity and balance growth with defense. Overall, available evidence indicates that PGPR-induced epigenetic changes represent a promising and environmentally friendly approach to crop protection; however, field-level validation and mechanistic confirmation in non-model crop species remain necessary before this strategy can be considered practically applicable.

## 1. Introduction

Plant diseases caused by pathogenic fungi and oomycetes pose a significant risk to global food production, with over 8000 species identified as pathogens [1]. The Irish potato famine, caused by *Phytophthora infestans*, resulted in widespread death and migration, while wheat stem rust (*Puccinia graminis*) remains a global threat to wheat productivity [2,3]. As observed throughout human history, pathogens such as rusts, powdery mildews, and oomycete *Phytophthora* species can infect numerous plants, reducing agricultural yields and crop quality. According to research published in Nature Ecology and Evolution, the Food and Agriculture Organisation of the United Nations (FAO) estimates that plant diseases cost the global economy approximately $220 billion annually, with 20–40% of crop production lost to pests [4]. A global study demonstrates that losses in five major crops (wheat, rice, maize, potato, and soybean) are 21.5% in wheat, 21.4% in soybean, 17.2% in potato, 22.5% in maize, and 30.0% in rice [4]. Invasive insects alone contribute at least USD 70 billion in additional losses globally. These impacts are compounded by climate change, which is expanding the geographic range and severity of many plant pathogens and pests. Official FAO data also indicates that up to 40% of crops are lost annually due to plant pests and diseases [5].

**Figure 1:**
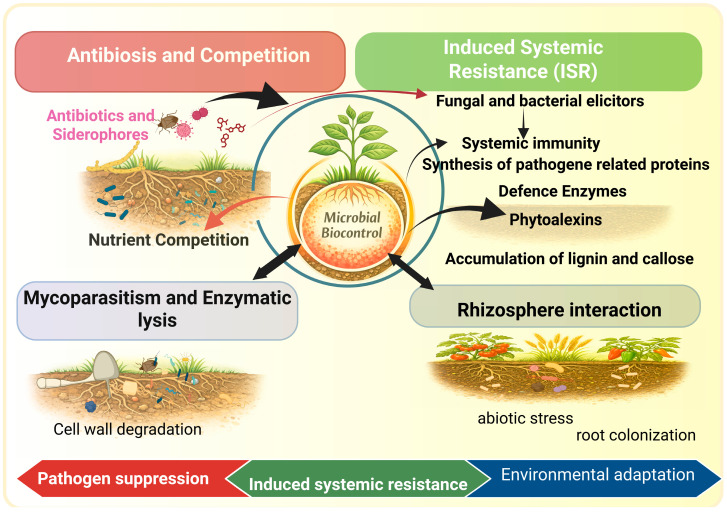
Overview of plant growth-promoting rhizobacteria (PGPR)-mediated biocontrol mechanisms in the rhizosphere. PGPR suppress pathogens through antibiosis, siderophore production, nutrient competition, and mycoparasitism, while simultaneously activating induced systemic resistance (ISR). This activation promotes the synthesis of defense proteins, phytoalexins, and the reinforcement of structural barriers such as lignin and callose, ultimately contributing to plant adaptation and enhanced resistance under environmental stress conditions. Created in BioRender Unal, D. (2026) https://BioRender.com/7zhuxlv (accessed on 16 April 2026).

Traditional methods have relied on resistant plant varieties developed through breeding and on chemical fungicides. Chemical fungicide application, while practical and effective, carries risks to human and animal health, and excessive use causes environmental damage. Concerns regarding their contribution to environmental contamination, seasonal restrictions, and the withdrawal of certain compounds from the market have led to strict regulations on their use. Biological control is one of the most studied strategies. Knowledge of plant–microorganism interactions is crucial for enhanced and more green disease control practices and interventions. Biological control agents (BCAs) are microorganisms inhibiting plant pathogens through molecular, physiological, and ecological means (see Figure 1). Plant Growth-Promoting Rhizobacteria (PGPR) have received significant attention from BCAs as eco-friendly substitutes for synthetic pesticides. PGPR naturally colonize the rhizosphere and endosphere and establish symbiotic relationships with host plants [6,7,8]. For example, endophytic bacteria like *Bacillus subtilis* enhanced plant growth and symbiosis, but have also been proven to suppress root rot caused by *Fusarium solani* under salt stress conditions [9]. PGPR can use several strategies and applications to improve plant health and disease resistance. These responses are mainly through the generation of antimicrobial compounds, competition for nutrients and space, and by affecting plants with systemic resistance via epigenetic pathways [10,11]. PGPR have been suggested to influence plant chromatin structure and gene expression patterns and may contribute to sustained defense-associated responses in key defense signaling pathways including salicylic acid (SA) and jasmonic acid/ethylene (JA/ET) pathways [10,11]. This shows that microbial-mediated immune modulation is even broader than classical signaling networks and extends into chromatin-level regulation, which is an important aspect of plant–microbe interaction. PGPR could exert its effects on plant defense mechanisms not only by generating antimicrobial agents but also through DNA methylation, histone modifications, and non-coding RNA regulation, which leads to a primed state that can lead to faster and stronger responses to future pathogen attacks [12,13].

Despite growing interest in PGPR-mediated plant protection, existing reviews have largely focused either on the biochemical mechanisms of PGPR biocontrol or on epigenetic regulation in plant immunity as separate topics. A comprehensive synthesis that explicitly connects PGPR-derived molecular signals to chromatin-level reprogramming and the establishment of immune memory has not been provided. Moreover, the extent to which PGPR-induced epigenetic states contribute to induced systemic resistance, and whether such states may persist across plant generations, remains poorly understood and underexplored in the literature. The present review addresses this gap by integrating current knowledge on PTI signaling, SAR and ISR pathways, bacterial elicitor recognition, and epigenetic mechanisms—including DNA methylation, histone modifications, and non-coding RNAs—within a unified framework. Unlike previous reviews, this work specifically focuses on how microbial signals reshape chromatin architecture at defense gene loci and discusses the translational relevance of these findings for sustainable crop protection.

## 2. Pattern-Triggered Immunity (PTI)

Plant growth-promoting rhizobacteria (PGPR) constitute a functionally and taxonomically diverse group of soil bacteria that colonize the plant rhizosphere and exert beneficial effects on plant growth, health, and stress tolerance [6,7,8]. The most extensively studied PGPR genera include *Bacillus*, *Pseudomonas*, *Rhizobium*, *Paenibacillus*, *Azospirillum*, and *Burkholderia*. These bacteria employ a range of direct and indirect mechanisms to promote plant fitness, including biological nitrogen fixation, phosphate solubilization, phytohormone production, siderophore secretion, and the suppression of soil-borne pathogens through antibiosis and competition [10,11]. Beyond these well-characterized growth-promoting functions, PGPR are increasingly recognized as potent inducers of plant immune responses through epigenetic and chromatin-level mechanisms, as discussed in subsequent sections. A summary of the major PGPR groups, representative species, and their principal plant-beneficial mechanisms is provided in Table S1.

Pattern-triggered immunity (PTI) is the first line of plant defense, recognizing and restricting pathogenic agents through detection of conserved microbe-associated molecular patterns (MAMPs/PAMPs) by plasma membrane-localized pattern recognition receptors (PRRs) [14,15]. Plants respond to PAMP perception with a series of multi-layered signaling cascades. Such mechanisms comprise acute cytoplasmic calcium elevation by opening channels and release from intracellular stores, which recruits calcium-dependent protein kinases (CDPKs) [16,17]; generation of reactive oxygen species (ROS) through the phosphorylation of NADPH oxidase RbohD via BIK1 [18,19]; and mitogen-activated protein kinase (MAPK) cascades involving the phosphorylation of MAPKKKs, MAPKKs, and MAPKs sequentially [20,21]. These signaling events converge on transcriptional reprogramming, thus triggering the activation of defense-related transcription factors within the WRKY, ERF, MYB, and bZIP families [22]. PTI products are accumulation of pathogenesis-related (PR) proteins such as chitinases and glucanases, synthesis of antimicrobial secondary metabolites (phytoalexins), and the physical reinforcement of cell wall structure via the deposition of callose and lignin-like cross-linking [22,23]. Of note, PTI features temporal waves of gene activation—from immediate-early responses within minutes to sustained defense over hours to days—establishing the transcriptional context in which epigenetic priming occurs.

## 3. Systemic Immunity

Plant immunity extends beyond localized defensive mechanisms to encompass intricate systemic processes that safeguard the entire organism over an extended duration. These systemic defensive mechanisms enhance plants resilience to subsequent pathogen infections, even in tissues distant from the initial site of infection. Systemic acquired resistance (SAR) and induced systemic resistance (ISR) are two main types of systemic immunity that have been studied extensively. The two types vary in their triggering mechanisms, signal transmission methods, and molecular composition [11].

### 3.1. Systemic Acquired Resistance (SAR)

Systemic Acquired Resistance (SAR) is a well-characterized broad-spectrum defense mechanism in plants, active against diverse pathogen classes. First described in the 1960s, SAR is now recognized as a critical component of plant immunity that can persist for weeks to months after the initial pathogen encounter. SAR is characterized by complex gene expression patterns of proteins that depend on the synchronized behavior of PR proteins which collectively confer broad-spectrum disease resistance. The initiation of SAR requires prior activation of PTI or ETI in response to pathogen invasion. Pathogen infection triggers the biosynthesis and systemic dissemination of signaling molecules to distal tissues, initiating defense responses [24]. Consequently, plants may be better protected against a broad range of diseases, as primed immune systems mount more rapid and robust responses to subsequent pathogen attacks. The molecular signaling mechanisms of SAR have been described extensively in *Arabidopsis* and other model organisms, revealing a plethora of signaling factors and regulatory cascades.

**Figure 2:**
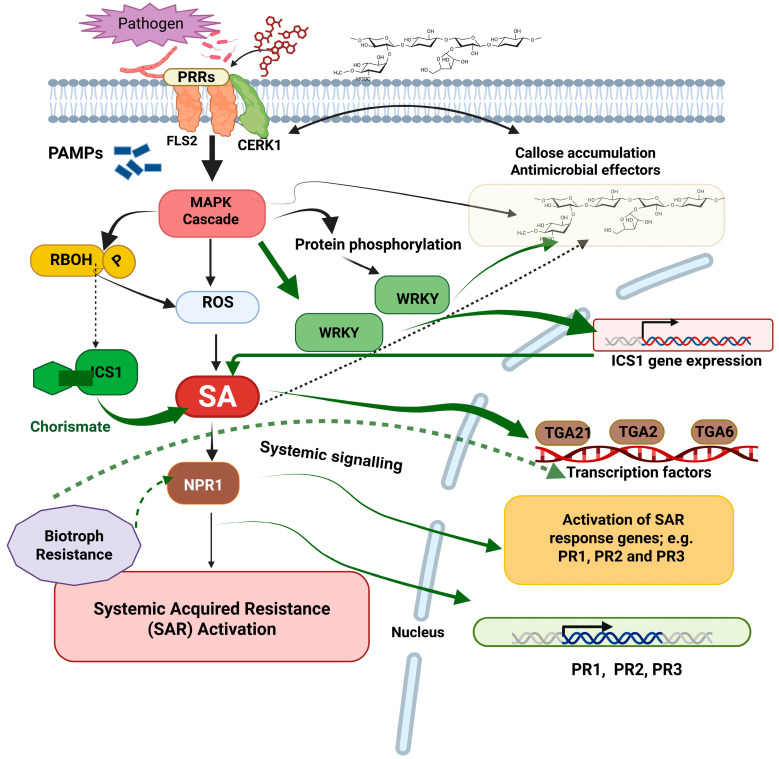
Salicylic acid-mediated signaling pathway in systemic acquired resistance (SAR). Upon pathogen perception, SA is synthesized primarily via the ICS1 pathway and accumulates in both infected and distal tissues. SA promotes the conversion of NPR1 oligomers into active nuclear monomers, which recruit TGA transcription factors to activate PR gene expression (PR-1, PR-2, PR-3). Long-distance SAR signaling is further supported by mobile metabolites, including methyl salicylate (MeSA), azelaic acid, glycerol-3-phosphate (G3P), and N-hydroxypipecolic acid (NHP), thereby establishing systemic priming and broad-spectrum resistance. Created in BioRender Unal, D. (2026) https://BioRender.com/pxr9wy9 (accessed on 16 April 2026).

The SAR pathway centers on salicylic acid (SA) as a primary mediator molecule, predominantly from the isochorismate pathway via ICS1 [25,26]. NPR1, as an SAR master regulator, is exposed to SA-dependent redox conversion from inactive cytoplasmic oligomers to active nuclear monomers, where it serves as a transcriptional co-activator by recruiting TGA transcription factors to activate PR gene expression [27,28] (Figure 2). PR effectors (for example, PR-1 [SAR marker], PR-2 [β-1,3-glucanase], and PR-3 [chitinase]) accumulate in systemically resistant tissues and directly influence pathogen restriction [29,30,31]. Other transcription factors such as WRKY18/40/60 and the recently identified SARD1/CBP60g have also been shown to regulate SA biosynthesis and defense gene expression [32,33,34]. SAR signal transduction proceeds by a network of pathways, which combines local SA accumulation with long-distance systemic signaling [35,36]. Some SA derivatives and mobile metabolites, such as methyl salicylate (MeSA), azelaic acid, glycerol-3-phosphate (G3P), and N-hydroxypipecolic acid (NHP) play important roles in long-range SAR signaling by leading to systemic SA accumulation and establishment of NPR1-mediated immunity through positive feedback loops [37,38]. The SAR signal transduction leads to cascading transcriptional activation—from rapid-early gene expression within hours to sustained defense gene expression over days [39,40,41,42]. Importantly, SAR underlies the primed state that promotes faster and stronger immune responses to subsequent pathogen exposures as opposed to constitutive defense-mediated activation. The priming requires accumulating inactive signaling structures, increased pattern recognition receptor (PRR) expression, and epigenetic processes aiding in the fast-reactivation of the gene [43]. Section 5 will discuss the epigenetic basis of SAR priming.

### 3.2. Induced Systemic Resistance (ISR)

**Figure 3:**
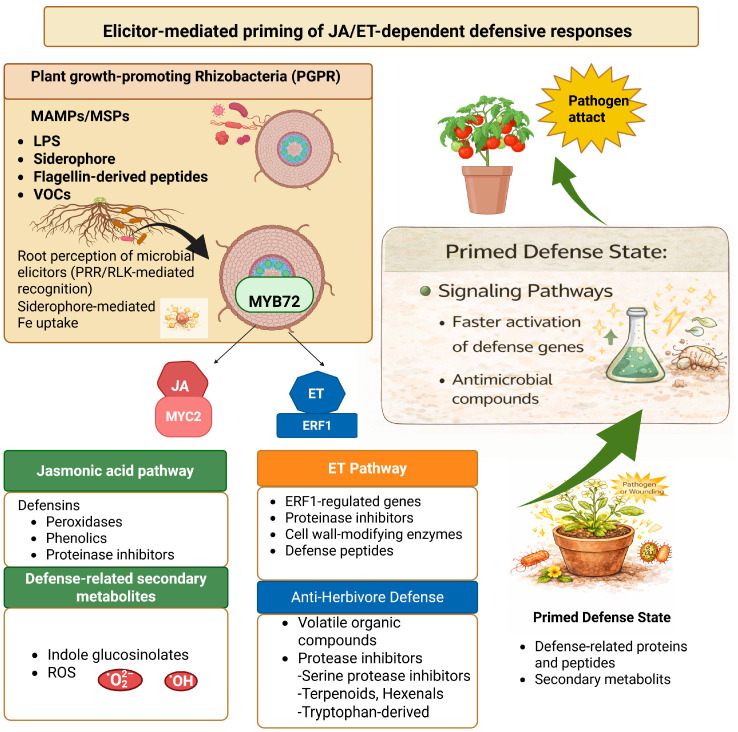
Elicitor-mediated priming of JA/ET-dependent defensive responses by PGPR. PGPR-derived elicitors, including flagellin, lipopolysaccharides, cyclic lipopeptides, and volatile organic compounds (e.g., 2,3-butanediol and acetoin), are perceived by plant pattern recognition receptors (PRRs), activating MAPK cascades and ROS production. These signals prime JA/ET signaling pathways by sensitizing key transcription factors MYC2, ERF1, and ORA59 without triggering full defense activation. Upon subsequent pathogen challenge, the primed state enables faster and stronger defense gene expression, thereby conferring broad-spectrum induced systemic resistance (ISR). Created in BioRender Unal, D. (2026) https://BioRender.com/asganmr (accessed on 16 April 2026).

Beneficial rhizobacteria (PGPRs) elicit induced systemic resistance (ISR), which confers broad-spectrum protection against fungi, bacteria, and viruses without directly killing pathogens [11]. Unlike SA-dependent SAR, ISR primarily operates through jasmonic acid (JA) and ethylene (ET) signaling pathways, although considerable crosstalk exists between these pathways [44,45]. Plant growth-promoting rhizobacteria have been widely reported to enhance plant tolerance to abiotic stresses such as salinity and drought through modulation of phytohormone signaling, antioxidant systems, and metabolic reprogramming, thereby contributing to ISR [9]. In line with this, endophytic *Bacillus subtilis* strains have been demonstrated to suppress *Fusarium solani*-induced root rot in chickpea under saline conditions by producing siderophores, cell wall-degrading enzymes, and indole-3-acetic acid, suggesting that PGPR-mediated disease suppression and stress tolerance may operate through overlapping signaling mechanisms [46]. Indeed, salt-tolerant PGPR strains have been shown to maintain their plant growth-promoting and biocontrol functions even under saline conditions, largely through the production of ACC deaminase, exopolysaccharides, and osmoprotectants that synergize with ISR-related defense pathways [46]. ISR enhances resistance not through elevated JA/ET production but through sensitization to these hormones, regulated by key transcription factors including MYC2, ERF1, and ORA59 [47,48]. Transcriptomic profiling of *Arabidopsis* roots colonized by *P. simiae* WCS417 has revealed distinct transcriptional signatures during ISR establishment, including the suppression of local immune outputs to facilitate beneficial colonization [49]. The defining feature of ISR is defense priming: the buildup of inactive signaling components and enhanced responsiveness of JA/ET pathways enables faster and stronger defense activation upon subsequent pathogen challenge, without the metabolic costs of constitutive defense [50] (Figure 3).

## 4. Bacterial Elicitors and Recognition

ISR elicitation depends on the recognition interaction between plant roots and beneficial bacteria; it involves detection of bacterial elicitors that prime immune readiness without triggering full defense activation [50]. Unlike effector-triggered immunity (ETI), which relies on intracellular NLR receptors to detect pathogen effectors and typically culminates in a hypersensitive response, ISR is induced by beneficial microbe-derived elicitors at the root surface and leads to a primed but not constitutively active defense state. The plant–microorganism recognition system is characterized by the complex receptor–ligand interactions occurring at the molecular level. Among the key PRRs implicated in ISR signaling, FLS2, BIK1, and PBL27 (Table 1) are well characterized for their roles in responding to conserved microbial structures and triggering defense signaling cascades. Beneficial bacteria secrete diverse elicitors including lipopolysaccharides, flagellin, siderophores, and volatile compounds. These molecules act as immune modulators that prime systemic resistance without triggering full defense activation. The nature of these interactions is determined by the source, amount, and degree of elicitation, as well as plant receptor sensitivity. This recognition is mediated by a complex network of molecular interactions that allows plants to discriminate between useful and pathogenic microorganisms within the rhizosphere. Lipopolysaccharides (LPS), components of the outer membrane of Gram-negative bacteria, are critical mediators of ISR elicitation, and structural diversity in LPS influences ISR induction efficiency [51]. Plant–bacterial interactions differ in structural diversity from one species to another because of variation in LPS morphology, and the LPS variants used are more potent at producing various forms of ISR. More recently, lipid A has been shown to play a prominent role in the elicitation of ISR from LPS as it is highly variable with modifications in its structure greatly affecting the plant response [52]. The surfactin-type lipopeptide produced by *B. subtilis* elicits plant defense responses and ISR when applied to cultured tobacco cell suspensions at micromolar concentrations, promoting early defense activities such as extracellular medium alkalinization, ion fluxes, and reactive oxygen species production [53]. Cyclic lipopeptides of beneficial rhizobacteria like *Pseudomonas* and *Bacillus* are the significant bioactive intermediate metabolites. They have antimicrobial activities, induce systemic resistance in plants and participate in the development of various ecological characteristics, ranging from movement and biofilm formation to root colonization [54]. Flagellin and other bacterial surface proteins may serve as ISR elicitors, including through the same receptors that mediate PTI responses, although they elicit different downstream outcomes [55]. Flagellin receptor FLS2 (FLAGELLIN SENSING 2) is known for performing dual roles in pathogen detection and mediating responses to beneficial bacteria [56]. The complex communication between plant-bacteria can also be demonstrated by the interaction between other bacterial surface proteins that trigger ISR via pattern recognition receptors [57], such as type III secretion system components and outer membrane proteins. Volatile organic compounds synthesized by PGPR, including 2,3-butanediol and acetoin, can induce ISR through air transmission, suggesting that direct physical contact of bacteria with roots is not required for ISR establishment. For the first time, 2,3-butanediol bioactivity was assessed using root application to *Arabidopsis*, where *B. subtilis* GB03 induced resistance against *Erwinia carotovora* [58]. These volatile compounds can be detected by plant roots at a distance from the bacterial source when released into soil air spaces. Recent research has identified additional volatile signaling molecules, including dimethyl disulfide and various terpenes, as mediators of long-distance communication between plants and bacteria [59]. Bacterial volatiles are proposed to be detected by putative chemoreceptors within plant roots, and it has been suggested that detection of these molecules at low concentrations may trigger signaling cascades that activate ISR, although the molecular identity of such receptors remains to be established (Table 1). Siderophores and other bacterial metabolites regulate iron homeostasis and root colonization, facilitating ISR promotion in a favorable environment for continual plant–microbe interaction [60]. Siderophore-bound iron enhances iron availability in the plant, and siderophore complexes may also function as signaling molecules acting as defense markers [61]. PGPR-produced siderophores can be detected by plants, which consequently mount a defense response against pathogens by modulating the immune system (Figure 3) [62]. Certain bacterial metabolites, such as cyclic lipopeptides and phenazines, also serve as major ISR factors, which can play a synergistic role with siderophores that promotes beneficial plant–microorganism relationships [63,64]. The identification of bacterial elicitor molecules by plants occurs through complex molecular mechanisms that enable discrimination between beneficial and pathogenic bacteria [57]. This discrimination process depends on multiple factors. These include signal concentration, timing of perception, duration of exposure, and physiological context. Beneficial bacteria generally promote a primed immune state rather than triggering full activation of plant defenses. Plant developmental stage and nutritional status also influence elicitor perception. Experimental evidence indicates that younger plants often show increased sensitivity to ISR-inducing bacterial signals [44]. The temporal dynamics of elicitor release represent an additional determinant. Sustained, low-level exposure to bacterial signals tends to favor ISR establishment, whereas rapid and high-intensity signaling is more commonly associated with direct defense activation. Recognition of bacterial elicitors is mediated by receptor-like kinase (RLK) and receptor-like protein (RLP) families, which differentiate among diverse bacterial molecular patterns [65]. These receptors function within multi-protein complexes that include co-receptors and adaptor proteins. Together, these components shape downstream immune signaling outcomes. Within this recognition framework, elongation factor Tu receptor (EFR) and peptidoglycan recognition protein (PGRP) families display differential ligand specificity and have been proposed to contribute to discrimination between pathogenic and beneficial bacteria, though direct evidence in the context of ISR remains limited [66].

Recent studies also suggest that root hair syntaxins and small RNAs participate in PGPR–plant communication, providing further insight into ISR establishment mechanisms [58,59]. Root hair syntaxins have been suggested to participate in membrane fusion processes that may facilitate bacterial signal perception, though their specific role in PGPR recognition requires further experimental confirmation. Small RNAs, including microRNAs and small interfering RNAs, act as modulators of gene expression linked to bacterial recognition and ISR signaling. These regulatory layers support context-dependent tuning of ISR responses according to microbial identity and environmental conditions. The principal bacterial elicitors of ISR and their recognition pathways are summarized in Table 1.

## 5. Epigenetic Mechanisms in Plant–PGPR Interactions

**Figure 4:**
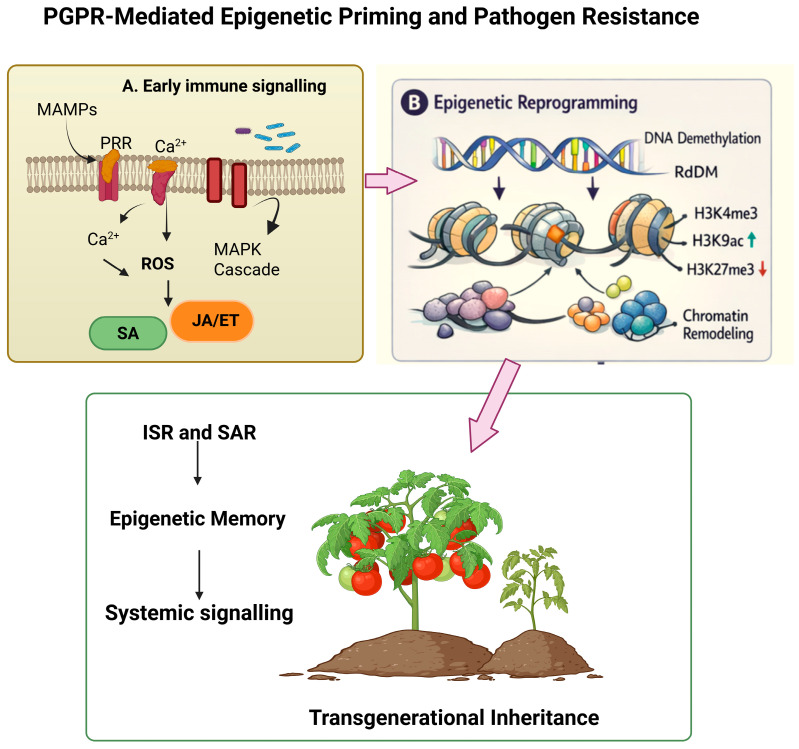
PGPR-mediated epigenetic priming and pathogen resistance through chromatin remodeling. PGPR colonization triggers convergent epigenetic reprogramming at defense loci through three interconnected layers: (1) DNA methylation dynamics, including ROS1-mediated promoter hypomethylation at defense regulators and RdDM-directed de novo methylation at transposable elements; (2) histone post-translational modifications, including enrichment of activating marks (H3K9ac, H4K5ac, H3K4me3) and removal of repressive marks (H3K27me3) at ISR-responsive genes; and (3) ncRNA-mediated regulation, involving siRNA-guided chromatin-modifying complexes and lncRNA-mediated recruitment of histone-modifying enzymes. Together, these modifications establish a transcriptionally primed chromatin state that enables faster and stronger defense gene activation upon subsequent pathogen challenge. Created in BioRender Unal, D. (2026) https://BioRender.com/lhx8jax (accessed on 16 April 2026).

Plant–PGPR interactions are increasingly associated with chromatin remodeling processes that operate alongside canonical immune signaling pathways [11,67,68,69]. The root surface-based detection of PGPR MAMPs triggers MAPK signaling, ROS accumulation, and reorganization of phytohormone networks, resulting in transcriptional activation of defense-related genes [11,67,68]. A growing body of evidence suggests that these signaling outputs are stabilized through epigenetic mechanisms (Figure 4). Indeed, chromatin-based mechanisms have been recognized as central mediators of environmental stress memory in plants, allowing somatic retention of adaptive responses and, in limited cases, potentially transgenerational effects; however, evidence for heritability remains context-dependent [70,71]. Chromatin accessibility at defense loci is regulated through DNA methylation dynamics, histone post-translational modifications, and ncRNA-mediated targeting, collectively establishing a transcriptionally primed state that facilitates enhanced immune activation upon subsequent pathogen challenge (Table 2) [72,73,74,75,76,77,78,79].

Table 2 demonstrates that DNA methylation turnover and histone acetylation are predominant epigenetic markers linked to PGPR-mediated immune priming, aligning with prior studies that illustrate chromatin-based stress memory and transcriptional responsiveness in plant defense mechanisms [69,72,97]. A significant bias towards model plants is apparent, with *Arabidopsis* and *Medicago* constituting the majority of experimental systems, underscoring a deficiency in crop-level validation and translational relevance [43]. Nodulation-associated epigenetic markers are mechanistically distinct from ISR-associated chromatin alterations, indicating context-dependent regulatory systems functioning in symbiotic and defensive interactions [80]. Additionally, ncRNA-mediated regulation functions as a versatile regulatory layer that integrates transcriptional and post-transcriptional control, thereby connecting microbial perception with chromatin accessibility and the activation of defense genes [75]. These patterns collectively highlight that PGPR-induced epigenetic reprogramming is complex and mechanistically diverse, emphasizing the necessity for comparative and field-based research to elucidate strain-specific and host-specific epigenetic responses.

### 5.1. DNA Methylation

DNA methylation is an important epigenetic mechanism regulating plant immune plasticity, which plays a role in CG, CHG, and CHH sequence environments, where genome integrity and transcriptional regulation are integrated [76,98]. Addition of a methyl group (5mC) to cytosine residues regulates developmental processes, stress adaptation, and immune responses [98]. De novo methylation is defined by the RNA-directed DNA methylation (RdDM) pathway mediated by plant-specific RNA polymerases Pol IV and Pol V, wherein siRNAs escort DRM2 methyltransferase to a target genomic region and play a role in epigenetic priming of defense genes [76,98,99,100]. Functional studies have confirmed the role of AGO4 in this pathway, as mutants showed greater susceptibility to pathogens [76]. Active demethylation is accomplished by DNA glycosylase/demethylases ROS1, DME, DML2, DML3, which remove the cytosine methylation marks [78,101]. ROS1 activation at the promoter of defense genes facilitates recruitment of WRKY transcription factors and enhances rapid transcriptional activation when the plant perceives the attacker as a pathogen [77,78]. PGPR colonization, such as *Pseudomonas simiae* WCS417 and *P. fluorescens*, has been associated with promoter hypomethylation in loci encoding defense regulators, auxin transporters, and secondary metabolite biosynthetic enzymes in root tissues. These alterations facilitate transcription factor availability and enable chromatin opening at stress-responsive sites [93]. Beyond these examples, DNA methylation reprogramming occurring at the molecular level during PGPR–plant interactions has been especially well characterized in legume symbioses. DNA methylation reprogramming of *Medicago truncatula* DNA is crucial in the host during Rhizobium interaction for nodule formation and development [80]. The DNA demethylase DEMETER (MtDME) is particularly overexpressed in the nodule differentiation area, as it de-methylates selected genomic regions to activate nodule key genes [80], like nodule specific cysteine rich proteins (NCR proteins), through dynamic demethylation that involves >1400 genes. Moreover, CHH hypermethylation at transposable elements (TEs) regulated by the RdDM pathway represses transposon activity and stimulates expression of symbiotic genes [81]. Apart from legume symbiosis, inoculation of *Phytolacca americana* with *Bacillus* sp. PGP5 and *Arthrobacter* sp. PGP41 mediated DNA methylation in roots that promoted plant development and function following microbiome inoculation [93]. Moreover, colonisation with *Bacillus subtilis* B26 significantly increased global DNA methylation in *Brachypodium distachyon* and in chronic drought stress, the growth of inoculated plants achieved higher global DNA methylation than in non-inoculated hosts, indicating potential epigenetic mechanisms for drought tolerance [95]. This plant–PGPR epigenetic interplay is in itself reciprocal: active DNA demethylation via the ROS1-dependent pathway is also required for mutualistic colonization in *Arabidopsis thaliana* with *Bacillus megaterium* YC4 [96]. On the mechanistic level, the demethylation of DNA controls the expression of genes encoding for myo-inositol biosynthesis and catabolism, and the encoded myo-inositol is a chemoattractant potentiating *B. megaterium* colonization against other rhizobacteria by the secreted myo-inositol in a selective manner, suggesting that plants can directly influence rhizosphere microbial community by epigenetic modulation [96].

### 5.2. Histone Modifications

Histone-modifying enzymes, including methyltransferases, demethylases, histone acetyltransferases, and histone deacetylases, are essential for the control of the immune response mediated by PGPR [67,102]. Remodeling of histone marks at defense promoters is a central epigenetic substrate of induced systemic resistance (ISR) [77,97]. PGPR strains such as *B. subtilis* and *P. fluorescens* have been reported to influence chromatin organization and may enhance transcriptional responsiveness of stress-related genes [67]. Cumulatively, activating acetylation marks, such as H3K9ac and H4K5ac, accumulate, stimulating primed chromatin formation to support rapid gene transcription during pathogen attack [67,77,87]. Histone acetyltransferases GCN5 and HAC1 contribute to euchromatin, HDACs such as HDA19 and HDA6 repress defense gene expression under basal conditions and maintain energy balance [67,103]. Histone methylation provides an additional layer of immune regulation. Deposition of H3K36me3 mediated by SDG8 promotes expression of jasmonate-responsive genes such as PDF1.2 and LOX2, while H3K4me3 deposition at these loci is regulated by distinct methyltransferases including SDG2 [67,78]. On the other hand, demethylases REF6 and JMJ705 remove repressive H3K27me3 marks and allow the activation of regulators such as NPR1, EDS1, and WRKY transcription factors [67,102]. Chromatin remodeling ATPases, BRM and SYD, also reposition nucleosomes allowing higher availability of promoters for transcription factors [67]. Eviction of histone variant H2A.Z by NRP1/2 chaperones further promotes full transcriptional initiation [104]. Transmissible histone marks, including H3K4me2, have been associated with intergenerational immune transmission in limited experimental contexts [99]. lncRNAs also play a role in this regulatory process by directing epigenetic enzymes toward target cell sites, or by serving as miRNA decoys to fine-tune defense responses [67,104]. A well-documented PGPR example involves *Paenibacillus polymyxa* CR1, which enhances systemic drought tolerance in *A. thaliana* by regulating histone modifications at the drought-responsive genes RD29A and RD29B [86]. Chromatin immunoprecipitation assays revealed that H3K9ac modifications are enriched in the coding region of RD29A, whereas H3K4me3 modifications are enriched in the coding region of RD29B, indicating gene-specific histone modification patterns that facilitate transcription of these defense genes under drought stress [86]. Knockout mutants lacking RD29A and RD29B showed greater susceptibility to drought and failed to develop induced systemic tolerance, confirming the functional importance of these PGPR-induced histone modifications [86]. At the metabolic level, acetyl-CoA serves as a key donor for histone acetylation, directly linking PGPR-induced metabolic changes to chromatin remodeling: PGPR have been shown to influence acetyl-CoA levels in plants, and when these levels increase, more acetyl groups become available for histone acetylation, promoting gene expression at growth-related loci [105,106,107].

### 5.3. ncRNA-Mediated Regulation

ncRNAs are important regulators of DNA methylation and histone modification dynamics [76,99]. miRNAs and siRNAs modulate nucleosome placement and chromatin architecture and affect gene expression at transcriptional and post-transcriptional levels [67,102]. Via RdDM, siRNAs direct DRM2 toward target defense loci and remodel its methylation landscape [67,78]. lncRNAs are often used as scaffolds to recruit chromatin remodeling complexes and histone modifying enzymes to specific genomic sites. One is ELENA1 which induces PR1 activation through displacement of FIBRILLARIN 2, and another is T5120 that enhances acetylation through sequestration of HDAC9 [67,107]. The siRNAs derived from transposons in association with AGO1 bind to SWI/SNF complexes, recruiting RNA polymerase II to distal defense genes for a primed transcriptional topology governed by the histone variant H2A.Z [107]. Upon pathogen challenge, eviction of H2A.Z (see Section 5.2) permits rapid transcriptional elongation [98,107]. MiR482/2118 miRNA families, for example, regulate NLR gene expression and generate phasiRNAs that prevent autoimmune activation in the absence of a pathogen [67,102]. ncRNAs have been proposed to contribute to cross-kingdom RNA interference via extracellular vesicles; however, mechanistic evidence and generality across plant–PGPR systems remain limited [78,103]. PGPR-induced ncRNA remodeling supports the generation of immune memory and growth–defense balance. For example, elevated miR396 during PGPR colonization supports developmental processes, while decreased miR393 strengthens auxin signaling [67,107]. lncRNAs like ALEX1 and T5120 additionally guide histone programming toward primed chromatin states [67,103]. Whether these changes contribute to transgenerational immune priming and improved performance of PGPR-treated progeny remains an open question that warrants further investigation [99,108]. At the level of small RNA regulation, PGPR interactions with leguminous plants have revealed specific miRNA roles in nodulation and symbiosis. In *Lotus japonicus* infected with rhizobia, miR171c targets NSP2, a transcription factor critical for nodule formation, while miR397 regulates copper homeostasis and serves as a marker for functional symbiotic nodules; its expression increases when nodules fix nitrogen and decreases in non-functional nodules [82]. In *Phaseolus vulgaris* infected with *Rhizobium etli*, miR172 regulates the APETALA2 transcription factor, thereby improving root growth, enhancing rhizobial infection, and increasing nitrogen fixation efficiency [83]. In *Arabidopsis*, *Bacillus amyloliquefaciens* FZB42 enhances plant defense by downregulating miR846, which regulates the JA signaling pathway and suppresses immune responses; suppression of miR846 activates defense genes and boosts resistance to pathogens [88]. Similarly, *Bacillus cereus* AR156 enhances immunity in *Arabidopsis* by suppressing miR825 and miR825*, thereby activating their target defense-related genes and increasing resistance to *Pst* DC3000 infection [109]. In *Medicago truncatula*, siRNA-producing Dicer-like (DCL) genes—including DCL2, DCL3, and DCL4—are significantly upregulated in nodules compared with other plant organs (MtDCL2b expression increases more than 20-fold in nodules relative to roots), strongly suggesting that siRNAs play a regulatory role in rhizobia–nodule symbiosis [84]. At the lncRNA level, *Bacillus subtilis* SL18r activates lncRNA MSTRG18363 in tomato, which inhibits miR1918 (a negative regulator of plant immunity), thereby indirectly promoting immune responses and enhancing resistance to *Botrytis cinerea* [90].

### 5.4. Crosstalk Among Epigenetic Mechanisms

DNA methylation, histone modifications, and non-coding RNAs form an interconnected regulatory framework governing plant immune responses [76,78]. The stability of epigenetic marks during cell division is supported by reciprocal interactions between histone and DNA methylation pathways: CMT3 recognizes H3K9me2 marks and contributes to maintenance of CHG methylation [78,107], while SUVH enzymes promote H3K9me2 accumulation in a DNA methylation-dependent context [107]. Conversely, the activating histone marks described in Section 5.2—including H3K9ac and H3K4me3—function as regulatory counterbalances that maintain defense genes in transcriptionally permissive chromatin states [78,110].

Non-coding RNAs further integrate these layers by guiding epigenetic modifiers to specific genomic loci. As outlined in Section 5.3, the trans-priming model incorporating TE-derived siRNAs, AGO1–SWI/SNF recruitment, and H2A.Z-mediated RNA polymerase II poising provides a mechanistic framework for coordinated epigenetic regulation [77,107]. Hormonal crosstalk involving salicylic acid and methyl jasmonate extends this regulatory network and has been associated with epigenetic memory formation, with some studies suggesting potential links to intergenerational effects [99,111]. The convergence of these three epigenetic layers—and their integration with hormonal signaling—establishes the chromatin architecture upon which PGPR-mediated immune priming operates, as discussed in Section 6 and Section 7.

## 6. MAMP/PAMP-Driven Chromatin Reprogramming

Pattern-recognition responses to pathogen- or microbe-associated molecular patterns (PAMPs/MAMPs) represent one of the earliest events driving immune activity in plants. Conserved microbial motifs, such as chitin and β-glucans, trigger pattern-triggered immunity (PTI) and activate related signaling cascades including MAPK modules, ROS bursts, and phytohormone biosynthesis [15]. Beyond their direct transcriptional outputs, these signaling networks are integrated with chromatin remodeling processes that mediate immune activation at the epigenetic level. As described in Section 5.2, MAMP/PAMP perception drives histone acetylation, demethylation of repressive marks, and H3K4me3 deposition, establishing a transcriptionally primed state at key immune regulators such as PR1 and WRKY70 [67,78,102]. These chromatin changes enable not only acute transcriptional activation but also the formation of a molecular memory that underlies accelerated secondary responses to subsequent pathogen challenge.

Transposable elements (TEs) constitute an additional regulatory dimension of chromatin during the immune response. ROS1-mediated demethylation of TEs positioned adjacent to defense genes is triggered upon pathogen perception, rendering these chromatin regions more accessible via flg22 signaling [73,112]. The resulting TE activation feeds into the trans-priming pathway described in Section 5.3, maintaining defense loci in a poised state permissive for rapid transcriptional reactivation [77,104].

PGPR colonization extends this MAMP/PAMP-driven chromatin readiness by introducing additional elicitor signals into the rhizosphere [113]. How these bacterial signals interface with the chromatin remodeling machinery described above—and how they ultimately establish ISR-associated epigenetic signatures—is discussed in detail in Section 7.

At the metabolic level, the primed chromatin state accelerates antimicrobial biosynthesis upon infection. Elevated flux through the aromatic amino acid pathway (Phe, Trp, Tyr) drives accumulation of hydroxycinnamic acid derivatives, flavonoids, and steroidal glycoalkaloids including α-tomatine—compounds that collectively restrict pathogen development [114,115,116,117,118,119]. Whether this chromatin-mediated metabolic pre-positioning extends to transgenerational immune priming in offspring of PGPR-treated plants warrants further investigation [99].

## 7. PGPR-Derived Elicitors and Epigenetic Priming

PGPR colonization is typically accompanied by the release of multiple elicitors—including lipopeptides, volatile organic compounds, flagellin fragments, and quorum-sensing molecules—that function as signaling cues contributing to immune preparedness. Building upon the chromatin readiness established through MAMP/PAMP signaling (Section 6), these bacterial elicitors activate ISR and SAR through pattern recognition receptors, initiating downstream MAPK signaling and ROS bursts that converge on epigenetic remodeling at defense-related loci [99]. In particular, PGPR-derived elicitors have been linked to enrichment of activating histone marks and promoter hypomethylation at immune genes, as described in Section 5.1 and Section 5.2 [67,77].

What distinguishes PGPR-mediated priming from basal MAMP/PAMP-driven chromatin reprogramming is its bidirectional and sustained nature. Unlike transient pathogen-triggered chromatin responses, PGPR colonization establishes a sustained epigenetic state through prolonged low-level elicitor exposure, supporting both defense competence and plant growth simultaneously [108,110]. Metabolomic analyses further suggest accumulation of hydroxycinnamic acids, flavonoids, benzoates, and steroidal glycoalkaloids—compounds that may contribute to biochemical barriers against pathogen invasion [114].

Unlike chemical priming agents such as BABA or MeJA, PGPR-mediated priming represents a biologically integrated process involving bidirectional communication between plant and microbe [108,110]. This integration is reflected in the growth–defense balance that characterizes PGPR-colonized plants: rather than constitutively activating defense pathways—which would impose metabolic costs—PGPR-induced epigenetic reprogramming maintains defense loci in a poised but not fully activated state.

The ncRNA-mediated regulatory adjustments described in Section 5.3—including the miR396/miR393 axis—provide one mechanistic perspective on how this balance is achieved, though whether these represent coordinated outputs of PGPR signaling or independent responses remains unresolved.

The degree to which PGPR strain identity, host species, and environmental context shape the specificity of epigenetic outcomes remains insufficiently characterized. Nevertheless, the overall regulatory pattern is consistent with a chromatin and hormonal environment that enhances defense competence without substantially compromising growth. Field-based validation of these mechanisms—including possible links to transgenerational immune preparedness—has yet to be demonstrated, and represents a critical priority for translating PGPR-mediated epigenetic priming into applied crop protection strategies [107].

It should be noted that the current literature on PGPR-mediated epigenetic priming contains several important contradictions and limitations that warrant explicit acknowledgment. First, strain-specificity is a recurring challenge: epigenetic outcomes reported for one PGPR strain are frequently not reproducible with closely related strains, and the molecular basis for these differences—whether rooted in elicitor profile, colonization efficiency, or host genotype interactions—remains poorly understood. For example, ISR-associated histone modification patterns documented for *Bacillus subtilis* strains differ substantially across studies, suggesting that generalizations across the *Bacillus* genus should be made with caution. Second, a persistent discrepancy exists between laboratory and field observations. Epigenetic priming effects that are robust and reproducible under controlled growth chamber conditions frequently attenuate or disappear entirely under field conditions, where soil complexity, competing microbiota, fluctuating environmental factors, and plant developmental heterogeneity introduce confounding variables that are difficult to control. Third, several mechanistic claims in the literature—particularly regarding transgenerational inheritance and cross-kingdom RNA transfer—are based on a limited number of studies, often conducted in single model systems, and have not been independently replicated. Collectively, these contradictions highlight the need for more rigorous, multi-environment, and multi-strain comparative studies before broad mechanistic conclusions can be drawn from the existing evidence base.

## 8. Future Perspectives

A critical limitation of the current evidence base must be acknowledged at the outset: the mechanistic framework described in this review derives predominantly from studies conducted in model plant systems, particularly *Arabidopsis thaliana* and *Medicago truncatula*. While these systems have been invaluable for dissecting molecular pathways, their translational relevance to cultivated crops and field conditions remains largely unvalidated. For the majority of PGPR strains and epigenetic mechanisms reviewed here, it is not yet established whether comparable chromatin reprogramming occurs in agronomically important species such as wheat, maize, soybean, or tomato under realistic field conditions. Advanced field will require a deliberate shift toward crop-centered and field-based research.

Several priorities can be identified for advancing this research area. Mechanistically, the relationship between microbial signaling and chromatin remodeling remains unclear. This limitation is particularly evident for strain-specific elicitor profiles and the epigenetic dimension of ISR–SAR crosstalk. Comparative analyses of histone modification patterns at shared regulatory loci may help clarify whether these pathways operate synergistically, competitively, or independently. Evidence in this area remains limited. The potential transfer of small RNAs across kingdoms via extracellular vesicles is also speculative and requires further investigation.

Technological progress will be important. Single-cell ATAC-seq and bisulfite sequencing applied to PGPR-colonized root tissues may provide spatial resolution beyond bulk-tissue approaches, enabling identification of cell populations undergoing chromatin reprogramming during ISR establishment. However, these approaches must be extended beyond *Arabidopsis* to crop species and, ultimately, to field-grown plants under agronomically relevant conditions. Integration of epigenomic, transcriptomic, metabolomic, and microbiome datasets across time-course experiments may further clarify relationships between upstream signaling and downstream metabolic outcomes.

The translational gap is particularly evident in three areas. First, epigenetic priming has been characterized almost exclusively under controlled laboratory conditions; how soil complexity, fluctuating temperatures, and biotic competition affect PGPR-induced chromatin states in the field remains unknown. Second, the persistence of epigenetic marks across plant developmental stages and growing seasons—a prerequisite for practical biocontrol application—has not been demonstrated in crop systems. Third, the potential for transgenerational immune priming, while suggested by preliminary evidence in wheat, requires validation across multiple crop species, PGPR strains, and field environments before it can be considered a reliable agronomic tool.

Targeted epigenome editing tools, including dCas9-based chromatin modifiers, offer promising avenues for testing causal relationships between specific epigenetic marks and defense phenotypes—and should be prioritized for application in crop species rather than model systems alone.

## 9. Conclusions

This review has synthesized current evidence positioning PGPR-induced epigenetic modulation as a mechanistically coherent and environmentally sustainable candidate strategy for enhancing plant immune competence. The convergent framework emerging from PTI, SAR, and ISR research demonstrates that PGPR colonization does not merely activate classical immune signaling pathways but also reprograms the chromatin landscape at defense-associated loci through three interdependent epigenetic layers. DNA methylation dynamics—particularly ROS1- and DEMETER-mediated demethylation at defense gene promoters and RdDM-directed CHH hypermethylation at transposable elements—establish a permissive chromatin context for transcriptional priming. Histone acetylation marks, including H3K9ac and H4K5ac deposited by GCN5 and HAC1, and methylation marks such as H3K4me3 and H3K36me3, collectively maintain defense loci in a poised state enabling rapid transcriptional reactivation upon pathogen challenge. Non-coding RNAs, including miRNAs, siRNAs, and lncRNAs, further refine this regulation by guiding chromatin-remodeling complexes to specific genomic loci and mediating the growth–defense balance during colonization.

Several important questions remain unresolved. The molecular specificity by which individual PGPR strains produce distinct elicitor profiles and thereby elicit specific epigenetic outcomes requires systematic comparative investigation. The possibility of transgenerational immune priming, suggested by preliminary evidence from wheat–Bacillus interactions, remains to be rigorously validated; current data are insufficient to establish heritability as a general feature of PGPR-mediated epigenetic responses. The extent to which ISR-associated and SAR-associated epigenetic signatures are orthogonal or convergent at shared regulatory loci remains unclear, particularly under conditions of simultaneous pathogen and beneficial microbial exposure. Addressing these questions through emerging single-cell epigenomic approaches, multi-omics integration across time-course colonization experiments, and targeted epigenome editing will be essential for translating mechanistic understanding into robust agricultural applications.

It should be noted that the majority of mechanistic evidence reviewed here derives from model plant systems, particularly *Arabidopsis thaliana* and *Medicago truncatula*, and that crop-level and field-based validation remains limited. Collectively, PGPR-induced epigenetic priming represents a biologically promising avenue for durable crop protection; however, its practical applicability will require further confirmation across diverse host species, PGPR strains, and agronomic conditions.

## Figures and Tables

**Table 1:** Bacterial Elicitors and Recognition Mechanisms in Induced Systemic Resistance.

Type	Bacterial Source	Recognition Mechanism & Plant Receptors	ISR Outcome	Ref.
Lipopolysaccharides (LPS)	Gram-negative PGPR strains	Structural recognition of lipid A component; pattern recognition without tissue necrosis induction. Receptors: Receptor-like kinases (RLKs); LPS-binding proteins; Pattern recognition receptors	Enhanced pathogen resistance; immune priming without full activation; systemic protection against diverse pathogens	[51,52]
Flagellin	Motile beneficial bacteria	Recognition of conserved flg22 peptide; context-dependent signaling differentiation from pathogenic flagellin. Receptors: FLS2 (FLAGELLIN SENSING 2); associated kinase complex	Differential immune outcomes compared to pathogen flagellin; priming activation; enhanced resistance to bacterial and fungal pathogens	[55,56]
2,3-Butanediol	*Bacillus subtilis* GB03; Volatile-producing PGPR	Volatile-mediated airborne signaling; root chemoreception at low concentrations. Receptors: putative root chemoreceptors; specialized volatile receptors	Long-distance ISR activation; systemic defense priming; pathogen resistance without direct contact requirement	[58,59]
Acetoin	*Bacillus subtilis* GB03; Metabolically active PGPR	Volatile compound perception; low-concentration detection mechanisms. Receptors: specific volatile receptors; chemosensory proteins	Enhanced defense gene expression; ISR establishment; improved plant growth and pathogen resistance	[58]
Siderophores	Iron-chelating bacteria	Iron homeostasis modulation; chemical signaling through iron availability. Receptors: iron transporters; signaling receptors; metal sensing proteins	Dual function: enhanced iron nutrition and defense signaling; improved plant fitness and pathogen resistance	[60,61]
Cyclic Lipopeptides	Biosurfactant producers	Membrane interaction and permeabilization; signal transduction through membrane dynamics. Receptors: membrane-associated receptors; lipid-sensing proteins	Direct antimicrobial activity; ISR enhancement; membrane-mediated defense activation	[54]
Bacterial Elongation Factor Tu	Various beneficial bacteria	Conserved protein recognition; beneficial bacteria discrimination from pathogens. Receptors: EFR (EF-Tu RECEPTOR); associated kinases	Beneficial bacteria discrimination; immune priming; context-dependent immunity modulation	[65,66]
Volatile Organic Compounds	Rhizosphere bacteria	Airborne chemical communication; long-distance signaling. Receptors: volatile detection systems; root chemoreception	Long-distance ISR signaling; enhanced plant growth; pathogen resistance through airborne communication	[59]

PGPR = Plant Growth-Promoting Rhizobacteria; ISR = Induced Systemic Resistance; FLS2 = FLAGELLIN SENSING 2; EFR = EF-Tu RECEPTOR; RLKs = Receptor-like kinases.

**Table 2:** PGPR-mediated epigenetic mechanisms involved in plant immunity and stress responses.

Section A: Symbiosis/Nodulation-Related Epigenetic Mechanisms				
PGPR (Bacterium)	Epigenetic Mechanism	Plant Species	Key Finding/Effect	Ref.
*Rhizobium*	DNA Methylation DNA Demethylation (MtDME)	*Medicago truncatula*	MtDME is expressed in the nodule differentiation zone, demethylating NCR gene regions; >1400 genes affected, N_2_ fixation supported	[80]
*Rhizobium*	DNA Methylation CG/CHG Demethylation + CHH Hypermethylation (RdDM)	*Medicago truncatula*	CG/CHG demethylation near NCR genes during late nodule development; CHH hypomethylation at transposons supports symbiotic gene expression	[81]
*Rhizobia*	Non-coding RNA—miRNA Regulation (miR171c & miR397)	*Lotus japonicus*	miR171c targets NSP2 (critical TF for nodule formation); miR397 regulates copper homeostasis; indicator of N2 fixation efficiency	[82]
*Rhizobium etli*	Non-coding RNA—miRNA Regulation (miR172)	*Phaseolus vulgaris*	miR172 regulates APETALA2 TF; root growth improved, rhizobial infection and N2 fixation efficiency enhanced	[83]
*Rhizobia (Medicago–Rhizobium)*	Non-coding RNA—siRNA (DCL2/DCL3/DCL4 ↑)	*M. truncatula*	mRNA levels of all DCL genes markedly elevated in nodules (MtDCL2b >20-fold); siRNAs may play a role in symbiosis and nodulation regulation	[84]
*Sinorhizobium fredii*	Histone Modification—H3K4me3	*Glycine max*	H3K4me3 plays an important role in nodulation by regulating gene expression in soybean root nodules	[85]
Sectin B: Defense/ISR-Related Epigenetic Mechanisms				
*Paenibacillus polymyxa* CR1	Histone Modification—H3K9ac & H3K4me3 Enrichment	*Arabidopsis thaliana*	H3K9ac enrichment at RD29A coding region, H3K4me3 at RD29B; activates defense genes for drought/salinity tolerance (IST)	[86]
*Paenibacillus* and *Bacillus*	Histone Modification—H3K4 Methylation & H3K9 Deacetylation (Priming)	*Arabidopsis thaliana*	PGPR mild signals establish histone modification marks in roots/leaves via hormonal pathways	[72,86]
*Bacillus subtilis* (PGPR)	Histone Modification—H3K9ac & H4K5ac	*A. thaliana*	*B. subtilis* application broadly increases H3K9ac and H4K5ac at stress tolerance (DREB2A) and auxin biosynthesis (YUCCA8) loci; prolonged histone changes establish epigenetic stress memory	[87]
*Bacillus amyloliquefaciens* FZB42	Non-coding RNA—miRNA Regulation (miR846 ↓)	*Arabidopsis thaliana*	FZB42 suppresses miR846, JA signaling activated, defense gene expression increases, pathogen resistance enhanced (ISR)	[88]
*Bacillus cereus* AR156	Non-coding RNA—miRNA Regulation (miR825/miR825* ↓)	*Arabidopsis thaliana*	AR156 suppresses miR825 and miR825*; target defense genes activated, resistance against Pst DC3000 enhanced	[89]
*Bacillus subtilis* SL18r	Non-coding RNA—lncRNA Regulation (MSTRG18363)	*Solanum lycopersicum* (tomato)	SL18r activates lncRNA MSTRG18363, suppresses miR1918, SlATL20 expression increases, resistance against *Botrytis cinerea* enhanced	[63,90]
*Bacillus amyloliquefaciens* LZ04	Non-coding RNA—lncRNA-miRNA-mRNA Regulatory Network	*A. thaliana*	LZ04 (via VOCs) modulates the lncRNA-miRNA-mRNA network, enhancing tolerance to high calcium stress and improving root growth	[91]
*Bacillus amyloliquefaciens* B16	DNA Methylation—PR1 & PR3 Promoter	*Triticum vulgare* (wheat)	In G0 plants primed with *B. amyloliquefaciens* B16, DNA methylation changes were observed at the PR1 and PR3 promoters, and this epigenetic memory was transmitted to the G1 and G2 generations	[92]
Section C: Symbiosis/Nodulation-Related Epigenetic Mechanisms				
*Bacillus* sp. PGP5/*Arthrobacter* sp. PGP41	DNA Methylation Change	*Phytolacca americana*	Inoculation-induced DNA methylation modifications enhance growth; effects persist after inoculant removal (epigenetic persistence)	[93]
*Burkholderia phytofirmans* PsJN	DNA Methylation Change	*Solanum tuberosum* (potato)	Alterations in DNA methylation patterns observed following beneficial endophyte colonization	[94]
*Bacillus subtilis* B26	DNA Methylation—Global Hypermethylation	*Brachypodium distachyon*	Colonization upregulates DNA methyltransferase expression; inoculated plants maintain higher global methylation levels under chronic drought stress	[95]
*Bacillus megaterium* YC4	DNA Methylation—Active DNA Demethylation	*Arabidopsis thaliana*	Secreted myo-inositol acts as a chemoattractant and biofilm inducer, selectively enhancing *B. megaterium* colonization	[96]
*Bacillus megaterium* YC4	DNA Methylation—Active DNA Demethylation (ROS1 pathway)	*Solanum lycopersicum* (tomato)	Conserved cross-species mechanism analogous to *Arabidopsis*; myo-inositol-mediated mutualism	[96]

## Data Availability

This review was conducted following a systematic literature search performed in PubMed, Web of Science, Scopus, and Google Scholar databases, covering publications up to March 2025. The following search terms and their combinations were used: “plant growth-promoting rhizobacteria”, “PGPR”, “epigenetics”, “DNA methylation”, “histone modification”, “non-coding RNA”, “induced systemic resistance”, “systemic acquired resistance”, “chromatin remodeling”, “plant immunity”, “ISR priming”, and “transgenerational immunity”. Articles were selected based on the following criteria: (i) peer-reviewed original research articles and reviews published in English; (ii) studies directly addressing epigenetic or chromatin-level mechanisms in plant–PGPR interactions; and (iii) studies on PTI, SAR, and ISR signaling relevant to the scope of this review. Book chapters and grey literature were excluded. Reference lists of selected articles were additionally screened to identify further relevant studies.

## Supplementary Materials

The following supporting information can be downloaded at: https://www.mdpi.com/article/10.3390/plants15091368/s1, Table S1. Major PGPR genera, representative species, key mechanisms, and plant benefits.

## Abbreviations

ABA	Abscisic Acid
ACC	1-Aminocyclopropane-1-Carboxylate
AGO	ARGONAUTE
ATAC-seq	Assay for Transposase-Accessible Chromatin with Sequencing
BABA	β-Aminobutyric Acid
BCA	Biological Control Agent
BIK1	Botrytis-Induced Kinase 1
BRM	BRAHMA chromatin remodeling ATPase
bZIP	Basic Leucine Zipper transcription factor
CBP60g	Calmodulin-Binding Protein 60g
CDPK	Calcium-Dependent Protein Kinase
CMT3	Chromomethylase 3
DCL	Dicer-Like protein
DML	DEMETER-Like demethylase
DME	DEMETER DNA demethylase
DRM2	Domains Rearranged Methyltransferase 2
dCas9	Catalytically dead Cas9
EDS1	Enhanced Disease Susceptibility 1
EFR	EF-Tu Receptor
ERF	Ethylene Response Factor
ET	Ethylene
ETI	Effector-Triggered Immunity
FAO	Food and Agriculture Organization
FLS2	Flagellin Sensing 2
G3P	Glycerol-3-Phosphate
GCN5	General Control Non-derepressible 5 histone acetyltransferase
H2A.Z	Histone variant H2A.Z
H3K4me2	Histone H3 Lysine 4 Dimethylation
H3K4me3	Histone H3 Lysine 4 Trimethylation
H3K9ac	Histone H3 Lysine 9 Acetylation
H3K9me2	Histone H3 Lysine 9 Dimethylation
H3K27me3	Histone H3 Lysine 27 Trimethylation
H3K36me3	Histone H3 Lysine 36 Trimethylation
H4K5ac	Histone H4 Lysine 5 Acetylation
HAC1	Histone Acetyltransferase 1
HDA/HDAC	Histone Deacetylase
ICS1	Isochorismate Synthase 1
ISR	Induced Systemic Resistance
IST	Induced Systemic Tolerance
JA	Jasmonic Acid
JMJ705	Jumonji Domain-Containing Protein 705
lncRNA	Long Non-Coding RNA
LOX2	Lipoxygenase 2
LPS	Lipopolysaccharides
MAMP	Microbe-Associated Molecular Pattern
MAPK	Mitogen-Activated Protein Kinase
MeJA	Methyl Jasmonate
MeSA	Methyl Salicylate
miRNA	MicroRNA
MYC2	MYC Transcription Factor 2
NCR	Nodule-specific Cysteine-Rich protein
ncRNA	Non-Coding RNA
NHP	N-Hydroxypipecolic Acid
NLR	Nucleotide-binding Leucine-rich Repeat receptor
NPR1	Nonexpressor of Pathogenesis-Related genes 1
NRP	Nucleosome Assembly Protein-Related Protein
NSP2	Nodulation Signaling Pathway 2
ORA59	Octadecanoid-Responsive Arabidopsis AP2/ERF 59
PAMP	Pathogen-Associated Molecular Pattern
PDF1.2	Plant Defensin 1.2
PGPR	Plant Growth-Promoting Rhizobacteria
PGRP	Peptidoglycan Recognition Protein
phasiRNA	Phased Small Interfering RNA
PR	Pathogenesis-Related protein
PRR	Pattern Recognition Receptor
PTI	Pattern-Triggered Immunity
RdDM	RNA-directed DNA Methylation
REF6	Relative of Early Flowering 6
RLK	Receptor-Like Kinase
RLP	Receptor-Like Protein
ROS	Reactive Oxygen Species
ROS1	Repressor of Silencing 1 demethylase
SA	Salicylic Acid
SAR	Systemic Acquired Resistance
SARD1	Salicylic Acid Induction Deficient 1
SDG	SET Domain Group methyltransferase
siRNA	Small Interfering RNA
SUVH	SU(VAR)3-9 Homolog
SYD	SPLAYED chromatin remodeling ATPase
TE	Transposable Element
TF	Transcription Factor
VOC	Volatile Organic Compound
WRKY	WRKY domain-containing transcription factor
