# Supplementary material for: PGPR-Mediated Plant Immunity: From Microbial Recognition to Epigenetic Priming
Source: Plants (Basel). 2026 Apr 30;15(9):1368. doi: 10.3390/plants15091368 (PMC13165268; doi:10.3390/plants15091368)
Supplement: Supplementary file 1 [file plants-15-01368-s001.zip › plants-4217063-supplementary.pdf]

Table S1. Major PGPR genera, representative species, key mechanisms, and plant benefits.

Genus	Representative Species	Key Mechanisms	Plant Benefits
Bacillus	*B. subtilis, B. amyloliquefaciens, B. velezensis, B. cereus*	Antibiosis, lipopeptide production (surfactin, iturin, fengycin), VOC emission, ISR induction, spore-forming biocontrol	Disease suppression, growth promotion, epigenetic priming of defense genes
Pseudomonas	*P. fluorescens, P. simiae WCS417, P. putida*	Siderophore production, 2,4-DAPG antibiotic secretion, LPS-mediated ISR elicitation, root colonization	Broad-spectrum disease resistance, ISR, root architecture improvement
Rhizobium* / *Sinorhizobium	*R. etli, S. fredii, R. leguminosarum*	Biological nitrogen fixation (nodulation), Nod factor signaling, DNA methylation reprogramming in host	Nitrogen nutrition, symbiotic gene activation, epigenetic regulation of nodule development
Paenibacillus	*P. polymyxa, P. alvei*	Antibiotic production (polymyxin), nitrogen fixation, phytohormone synthesis, histone modification-mediated IST	Drought/salinity tolerance, disease suppression, chromatin-level defense priming
Azospirillum	*A. brasilense, A. lipoferum*	Auxin (IAA) and gibberellin production, nitrogen fixation, root hair proliferation	Root architecture enhancement, nutrient uptake, stress tolerance
Burkholderia	*B. phytofirmans PsJN, B. vietnamiensis*	Endophytic colonization, ACC deaminase activity, DNA methylation reprogramming in potato, phytohormone modulation	Cold/stress tolerance, epigenetic modification of host gene expression, growth stimulation
